# False claims of equivalence in the neurosurgical trauma literature: prevalence and associated factors—a systematic review protocol

**DOI:** 10.1136/bmjopen-2020-044794

**Published:** 2024-07-30

**Authors:** André Luiz Freitas Oliveira Júnior, João Vitor Miranda Porto Oliveira, Angelos G Kolias, Wellingson S Paiva, Davi Jorge Fontoura Solla

**Affiliations:** 1 Bahiana School of Medicine and Public Health, Salvador, Brazil; 2 Division of Neurosurgery, University of Cambridge, Cambridge, UK; 3 Division of Neurosurgery, University of São Paulo, São Paulo, Brazil; 4 Department of Neurosciences and Behaviour Sciences, University of São Paulo, Ribeirao Preto, Brazil

**Keywords:** NEUROSURGERY, TRAUMA MANAGEMENT, Neurosurgery, Neurological injury, NEUROPATHOLOGY, Clinical trials

## Abstract

**ABSTRACT:**

**Introduction:**

Research quality within the neurosurgical field remains suboptimal. Therefore, many studies published in the neurosurgical literature lack enough statistical power to establish the presence or absence of clinically important differences between treatment arms. The field of neurotrauma deals with additional challenges, with fewer financial incentives and restricted resources in low-income and middle-income countries with the highest burden of neurotrauma diseases. In this systematic review, we aim to estimate the prevalence of false claims of equivalence in the neurosurgical trauma literature and identify its predictive factors.

**Methods and analysis:**

The Preferred Reporting Items for Systematic Review and Meta-Analyses recommendations were followed. Randomised clinical trials that enrolled only traumatic brain injury patients and investigated any type of intervention (surgical or non-surgical) will be eligible for inclusion. The MEDLINE/PubMed database will be searched for articles in English published from January 1960 to July 2020 in 15 top-ranked journals. A false claim of equivalence will be identified by insufficient power to detect a clinically meaningful effect: for categorical outcomes, a difference of at least 25% and 50%, and for continuous outcomes, a Cohen’s *d* of at least 0.5 and 0.8. Using the number of patients in each treatment arm and the minimum effect sizes to be detected, the power of each study will be calculated with the assumption of a two-tailed alpha that equals 0.05. Standardised differences between the groups with and without a false claim of equivalence will be calculated, and the variables with a standardised difference equal or above 0.2 and 0.5 will be considered weakly and strongly associated with false claims of equivalence, respectively. The data analysis will be blinded to the authors and institutions of the studies.

**Ethics and dissemination:**

This study will not involve primary data collection. Therefore, formal ethical approval will not be required. The final systematic review will be published in a peer-reviewed journal and presented at appropriate conferences.

**STRENGTHS AND LIMITATIONS OF THIS STUDY**

- This is the first systematic review to evaluate the prevalence of false claims of equivalence in the neurosurgical trauma literature and its associated factors.
- The knowledge generated from this study may inform recommendations to enhance the neurosurgical research quality.
- Due to the exclusion of non-randomised clinical trials and observational studies, as well as other journals that are not top ranked within the field, the results of this study may not be fully generalisable.
- But considering that randomised clinical trials, compared with other study designs, usually are better planned and conducted, besides being published in journals with more rigorous peer review, our results may be conservative and thus strengthened.

## Introduction

 Reviews have shown that the quantity and the quality of clinical trials in neurosurgery remain suboptimal.^1^,^4^ Among the main limitations, the following are highlighted: absence of sample size calculation, limited sample size, single-centre recruitment and incomplete subject follow-up.^2 3^ Therefore, many studies published in the neurosurgical literature lack enough statistical power to establish the presence or absence of clinically important differences between treatment arms.^12 5^,^9^

The field of neurotrauma deals with additional challenges. Compared with other neurosurgical subspecialties, there is less financial incentive for neurotrauma research development, in part due to its disproportionately higher incidence in low-income and middle-income countries (LMICs) compared with high-income countries (HICs). The highest burden of neurotrauma diseases lies in LMIC with about 90% of global injury-related deaths, but these countries tend to lack adequate resources for qualified scientific production.^10^ Paradoxically, most of the high-quality studies looking at traumatic brain injury (TBI) are funded and conducted by HIC institutions, and less than 5% of 6708 published reports had a LMIC affiliation.^11 12^ Few initiatives are in progress to improve global neurotrauma care and research, such as the Global Health Research Group on Neurotrauma, funded by the National Institute for Health Research.^11^

More than 50% of neurotrauma clinical trials have recruited less than 100 subjects, and the median total sample size is around 70 subjects.^3^,^5^ Besides a higher odd of false discoveries, insufficient statistical power is associated with false claims of equivalence.^3^ In this systematic review, we aim to estimate the prevalence of false claims of equivalence in the neurosurgical trauma literature and identify its predictive factors.

## Methods and analysis

### Protocol and registration

This systematic review and meta-analysis will be reported following the Preferred Reporting Items for Systematic Review and Meta-Analyses guidelines.

### Eligibility criteria

Published randomised clinical trials (RCTs) that enrolled only TBI patients and investigated any type of intervention (surgical or non-surgical) will be eligible for inclusion. The following journals were selected for screening based on the impact factor and importance to the neurosurgical trauma literature: *New England Journal of Medicine* (NEJM); *Lancet*; *Lancet Neurology*; *Journal of the American Medical Association* (JAMA); *JAMA Neurology*; *Journal of Neurology, Neurosurgery and Psychiatry* (JNNP); *Neurosurgery*; *Journal of Neurosurgery*; *Neurosurgical Focus*; *World Neurosurgery*; *Acta Neurochirurgica*; *Journal of Neurotrauma*; *Intensive Care Medicine*; *Critical Care*; and *Neurocritical Care*. For RCT with multiple publications, only the reporting of the primary outcome was considered. Considering that randomised clinical trials, compared with other study designs, usually are better planned and conducted, besides being published in journals with more rigorous peer review, we believe these criteria will strengthen our results.

### Information sources and search strategy

The MEDLINE/PubMed database will be searched for articles in English published from January 1960 to July 2020. The descriptors (((((Traumatic brain injury[Title/Abstract]) OR (TBI[Title/Abstract])) OR (Brain trauma[Title/Abstract])) OR (Brain concussion[Title/Abstract])) OR (Brain contusion[Title/Abstract])) OR (head trauma[Title/Abstract]) OR (head injury[Title/Abstract]) OR (brain injury[Title/Abstract]) will be used. The filter ‘Randomised clinical trials’ will be applied.

### Study selection

The search strategy aims to achieve a sample of RCTs published in the neurosurgical trauma literature from which negative trials could be selected. Negative trials will be defined as those that concluded equivalent outcomes (either dichotomous or continuous) in the treatment arms by explicitly stating it (eg, ‘there was no statistically significant difference between the groups’). Otherwise, positive trials will be used as controls. Only the primary outcome intention to treat analysis will be considered, identified by a clear statement of the authors in the methods section or a clear primary focus of the article. If no clear primary outcome can be identified, the neurological/functional outcome or death, hierarchically, will be considered the outcome of interest. All articles’ titles and abstracts were screened by two authors (ALFOJR and JVMPO) for eligibility. The selected articles will be adjudicated by a third author (DJFS), and disagreements will be resolved by consensus. Additional studies identified in the reference section of the selected articles can be included if the eligibility criteria are fulfilled. No RCT studies, as well as no TBI works, will be excluded.

### Data collection and analysis

Data will be abstracted and recorded on a standardised form regarding the following: journal, year of publication, study country (high income or low and middle income), first and last author affiliations (neurosurgery or other), the presence of a statistician among the authors, single-centre or multicentre trial, type of trial design (superiority, non-inferiority or equivalence), the presence of a priori sample size and power calculation, type of TBI (mild, moderate and severe), setting (prehospital or intrahospital), intervention (surgical, drug or other), allocation concealment and blinding, number of patients, follow-up period, the event rates in the two treatment arms, the presence of a post hoc power calculation, the discussion of lack of power as a limitation, funding (industry, independent or none) and conflict of interest (when explicitly stated). The full text of the included articles will be systematically reviewed by two authors (ALFOJR and JVMPO). The interobserver agreement and the *κ-*statistic will be calculated. Disagreements will be resolved by a third author (DJFS). The data analysis will be blinded to the authors and institutions of the study.

The primary outcome will be the prevalence of false claim of equivalence in neurosurgical literature. A false claim of equivalence will be identified by insufficient power to detect a clinically meaningful effect, which will be defined under two scenarios for each type of outcome as follows: for categorical outcomes, a difference of at least 25% and 50% between the two groups given the control group baseline event rate, and for continuous outcomes, a Cohen’s *d* of at least 0.5 and 0.8 (between groups) given the control group outcome values. Using the number of patients in each treatment arm and the minimum effect sizes to be detected, the power of each study will be calculated with the assumption of a two-tailed alpha that equals 0.05.

Traditional descriptive statistics will be used to present the included RCT characteristics. Standardised differences between the groups with and without a false claim of equivalence will be calculated as proposed by Yang and Dalton.^13^ The variables with a standardised difference equal or above 0.2 and 0.5 will be considered weakly and strongly associated with false claims of equivalence, respectively. All analyses will be conducted with the SPSS software (IBM Corp. SPSS Statistics for Windows, V.24.0. Armonk, NY).

### Risk of bias in individual studies

The entire text of each included paper will be evaluated in a structured fashion for prespecified attributes. The second version of the Cochrane risk-of-bias tool (RoB 2) for randomised trials will be used.^14^ RoB 2 is structured into a fixed set of domains of bias, focusing on different aspects of trial design, conduct and reporting. Within each domain, a series of questions (signalling questions) aim to elicit information about features of the trial that are relevant to risk of bias. A proposed judgement about the risk of bias arising from each domain is generated by an algorithm, based on answers to the signalling questions. Judgement can be ‘low’ or ‘high’ risk of bias or can express ‘some concerns’.

## Ethics and dissemination

This study will not involve primary data collection. Therefore, formal ethical approval will not be required. The final systematic review will be published in a peer-reviewed journal and presented at appropriate conferences. This protocol may be adapted for the analysis of other innovative surgical and invasive procedures.
