# Supplementary material for: False claims of equivalence in the neurosurgical trauma literature: prevalence and associated factors—a systematic review protocol
Source: BMJ Open. 2024 Jul 30;14(7):e044794. doi: 10.1136/bmjopen-2020-044794 (PMC11293374; doi:10.1136/bmjopen-2020-044794)
Supplement: Supplementary file 1 [file bmjopen-2020-044794.reviewer_comments.pdf]

PEER REVIEW HISTORY

BMJ Open publishes all reviews undertaken for accepted manuscripts. Reviewers are asked to complete a checklist review form (**http://bmjopen.bmj.com/site/about/resources/checklist.pdf**) and are provided with free text boxes to elaborate on their assessment. These free text comments are reproduced below.

ARTICLE DETAILS

**TITLE (PROVISIONAL)**	FALSE CLAIMS OF EQUIVALENCE IN THE NEUROSURGICAL TRAUMA LITERATURE: PREVALENCE AND ASSOCIATED FACTORS – A SYSTEMATIC REVIEW PROTOCOL
**AUTHORS**	Oliveira Júnior, André; Oliveira, João Vitor; Kolias, Angelos; Paiva, Wellingson; Fontoura Solla, Davi

VERSION 1 – REVIEW

**REVIEWER**	Griffith, Matthew<br>Dwight David Eisenhower Army Medical Center
**REVIEW RETURNED**	26-Oct-2020

**GENERAL COMMENTS**	The current protocol is very well written and a very important subject to explore. I love the idea of analyzing the current data with respect to study power. I recommend this protocol for publication.<br><br>There are minor grammatical errors to correct:<br><br>Page 7/16 (on the PDF)<br><br>Line 17: change “there is fewer….” to “there is less financial incentive for neurotrauma research…”<br><br>Line 24-25: Sentence beginning with Paradoxically should be changed to “Paradoxically, most of the high-quality studies looking at traumatic brain injury are funded and conducted….”<br><br>Page 9/16 (on the PDF)<br><br>The paragraph starting on line 8. The differences of at least 25% and 50% between the groups is confusing as is the cohen d of at least 0.5 and 0.8. Are those for each individual group, between groups?<br><br>Otherwise great study, keep up the good work.

**REVIEWER**	Garg, Kanwaljeet<br>All India Institute of Medical Sciences
**REVIEW RETURNED**	27-Oct-2020

**GENERAL COMMENTS**	This article is a protocol of a systematic review on false claims of equivalence in RCTs involving any medical or surgical treatment in patients with traumatic brain injury.<br>The topic of the article is very pertinent and will provide us new insight about

**REVIEWER**	Dillen, Kim<br>University of Cologne, Department of Palliative Medicine
**REVIEW RETURNED**	10-Feb-2021

**GENERAL COMMENTS**	Thank you for the invitation to review this interesting review protocol.<br><br>General:<br>• Check clinically meaningful effects vs. Statistically significant effects<br><br>Introduction:<br>• Part of the third paragraph could be moved up to the first paragraph<br>• rationale is not clear, please elaborate<br><br>Methods and Analysis:<br>• please explain why only RCTs and top-ranked journals were chosen<br>• in the search strategy the authors mention „clinical trail“ as search criteria while the text suggests that only RCTs were included, please clarify<br>• one database seems insufficient<br>• please explain the chosen cut-offs for the estimate of effect for both categorial and continuous outcomes and cite appropriate literature<br>• please elaborate on chosen outcomes and potential comparator groups as well as exclusion criteria<br>• please add full initials for the two authors (A and J) who screened and reviewed articles<br><br>References:<br>• please check for consistency

VERSION 1 – AUTHOR RESPONSE

- Reviewer 1
  - This is a great design and a great idea to explore. I only have a few minor corrections, but otherwise is a great start!
    - Response from authors: Thank you for your review!
  - Page 7/16 (on the PDF)
    - Line 17: change “there is fewer….” to “there is less financial incentive for neurotrauma research…”.
      - Response from authors: Changed.
    - Line 24-25: Sentence beginning with Paradoxically should be changed to “Paradoxically, most of the high-quality studies looking at traumatic brain injury are funded and conducted….”.
      - Response from authors: Changed.

- Page 9/16 (on the PDF)
    - The paragraph starting on line 8. The differences of at least 25% and 50% between the groups is confusing as is the cohen d of at least 0.5 and 0.8. Are those for each individual group, between groups?
        - Response from authors: Between groups. We also clarified this in the manuscript.
- Reviewer 2
    - This article is a protocol of a systematic review on false claims of equivalence in RCTs involving any medical or surgical treatment in patients with traumatic brain injury. The topic of the article is very pertinent and will provide us new insight about.
        - Response from authors: Thank you! We believe our study will provide some valuable insights regarding the recommendations to enhance the neurosurgical research quality.
- Reviewer 3
    - General:
        - Check clinically meaningful effects vs. Statistically significant effects.
            - Response from authors: We considered clinically meaningful effects.
    - Introduction
        - Part of the third paragraph could be moved up to the first paragraph.
            - Response from authors: To keep the "insufficient statistical power is associated with false claims of equivalence" part contextualized with the rest, we believe it is better to keep it close to the final sentence of our introduction, which clarifies the direction we're pathing in our study.
        - Rationale is not clear, please elaborate.
            - Response from authors: Considering that research quality within the neurosurgical field remains suboptimal, we hypothesize that these studies commonly have insufficient statistical power, which is associated with false claims of equivalence. Therefore, we aim to estimate the prevalence of false claims of equivalence in the neurosurgical trauma literature and identify its predictive factors. Studies with inferior designs and published in lower quality journals would probably present even worse results.
    - Methods and Analysis:
        - Please explain why only RCTs and top-ranked journals were chosen.
            - Response from authors: We explained this in last sentence from "Eligibility criteria". Studies with inferior designs and published in lower quality journals would probably present even worse results.
        - In the search strategy the authors mention „clinical trail" as search criteria while the text suggests that only RCTs were included, please clarify.
            - Response from authors: We clarified this in the text.
        - one database seems insufficient.
            - Response from authors: We selected only the research papers available on PubMed to standardize the selection, since all journals included are available on PubMed.
        - please explain the chosen cut-offs for the estimate of effect for both categorial and continuous outcomes and cite appropriate literature.
            - Response from authors: The cut-offs were chosen based on the Cohen's conventions for small, medium and large effects (available on https://doi.org/10.1080/03610911003650383).
        - please add full initials for the two authors (A and J) who screened and reviewed articles.

        - Response from authors: Added.
  - References:
    - please check for consistency.
      - Response from authors: Checked.

VERSION 2 – REVIEW

**REVIEWER**	Dillen, Kim<br>University of Cologne, Department of Palliative Medicine
**REVIEW RETURNED**	26-Mar-2021

**GENERAL COMMENTS**	Thank you for incorporating my comments into the current version of the protocol. It reads very well.
